# Glenoid version by CT scan: an analysis of clinical measurement error and introduction of a protocol to reduce variability

**DOI:** 10.1007/s00256-015-2207-4

**Published:** 2015-07-23

**Authors:** Fabian van de Bunt, Michael L. Pearl, Eric K. Lee, Lauren Peng, Paul Didomenico

**Affiliations:** VU University Medical Center, De Boelelaan 1117, 1081 HV Amsterdam, Netherlands; Kaiser Permanente, 4760 Sunset Blvd, Los Angeles, CA 90027 USA

**Keywords:** Glenoid, Version, 3D measurement, Version measurement, Shoulder, Glenoid version

## Abstract

**Objective:**

Recent studies have challenged the accuracy of conventional measurements of glenoid version. Variability in the orientation of the scapula from individual anatomical differences and patient positioning, combined with differences in observer measurement practices, have been identified as sources of variability. The purpose of this study was to explore the utility and reliability of clinically available software that allows manipulation of three-dimensional images in order to bridge the variance between clinical and anatomic version in a clinical setting.

**Materials and methods:**

Twenty CT scans of normal glenoids of patients who had proximal humerus fractures were measured for version. Four reviewers first measured version in a conventional manner (clinical version), measurements were made again (anatomic version) after employing a protocol for reformatting the CT data to align the coronal and sagittal planes with the superior-inferior axis of the glenoid, and the scapular body, respectively.

**Results:**

The average value of clinical retroversion for all reviewers and all subjects was −1.4° (range, −16° to 21°), as compared to −3.2° (range, −21° to 6°) when measured from reformatted images. The mean difference between anatomical and clinical version was 1.9° ± 5.6° but ranged on individual measurements from −13° to 26°. In no instance did all four observers choose the same image slice from the sequence of images.

**Conclusions:**

This study confirmed the variation in glenoid version dependent on scapular orientation previously identified in other studies using scapular models, and presents a clinically accessible protocol to correct for scapular orientation from the patient’s CT data.

## Introduction

Glenoid version is of interest in understanding normal shoulder biomechanics and pathological conditions inclusive of instability, arthritis, and developmental dysplasia. Normal glenoid version in most studies has been reported close to 0°, sometimes with slight anteversion but more often slight retroversion with values typically less than 10° in either direction [1–8]. Variance from normal version alters glenohumeral mechanics and may predispose to instability and arthropathy. In the prosthetic shoulder, deviation from the native version has been shown to increase stress and wear of the glenoid component [9–12]. These findings have led to the prevailing recommendation to strive for normalization or neutralization of glenoid version during shoulder arthroplasty [13, 14].

Concomitant with this, it has become clear that the accepted gold standard for measuring version, computed tomography (CT), presents numerous reliability issues. For one, version values measured at different heights along the superior/inferior axis of the glenoid have been shown to differ by several investigators, consistently finding more retroversion at superior positions [15–18]. However, the clinical literature has not been rigorous in defining the specifics of image slice selection nor the reliability with which it is made. The original recommendation to measure version at the mid glenoid level was less arbitrary when first described when a CT scan of the shoulder had three or four cuts that traversed the glenoid [1].

Besides these, version measurements are also highly dependent on other variables that alter scapular orientation. Some of these variables are arbitrarily determined by factors such as the patient position in the scanner and the orientation of the slice settings, and others relate to measurement practices by the examiner. It has been demonstrated that rotating a cadaveric scapula in its own coronal plane may alter version measured by CT scan by 12°, this variability also occurs when rotating the scapula in its sagittal plane [5, 19].

Glenoid version remains an important two-dimensional representation of the complex three-dimensional shape of the scapula but our understanding of what constitutes normal and the importance of deviation from normal requires thorough reconsideration. We undertook this study to explore the utility and reliability of clinically available software that allows manipulation of three-dimensional images in order to bridge the variance between clinical and anatomic version in a clinical setting.

## Materials and methods

With IRB approval, 22 CT scans of normal scapulae were taken from consecutive patients with proximal humerus fractures between September 2011 and June 2012. Two CT scans were considered suboptimal studies because the scan did not capture the entire scapula. These scans were removed from the subject pool leaving a study group of 20. Our study group existed of six males and 14 females, with a mean age of 77.0 years old. The CT scan parameters were as follows: slice thickness: 0.63 mm, pitch: 0.53, kVp: 140, mA: 300, focal spot: large, reconstruction algorithm: BONEPLUS. The CT scans were anonymized and measured for version by four independent reviewers (three musculoskeletal radiologists and one orthopedic surgeon specialized in shoulder surgery) using a protocol that was specific for slice selection and placement of reference axes.

Version measurements were made again after employing a protocol for reformatting the CT data that aligned the images with the scapular axes. A partitioned panel with three orthogonal views (corresponding to Fig. 3a) was utilized to compensate for the coronal obliquity of the scapula, its medial to lateral anteversion, and craniocaudal adduction/abduction, as it conforms to the underlying chest wall. The final ‘anatomic’ axial reformatted images of the scapula were obtained by constraining these confounding inclinations through the placement of crosslines along the body of the scapula in the sagittal thumbnail (coronal compensation), in the axial (scapular anteversion compensation), and coronal (abduction correction). The reviewers were blinded to their earlier measurements and to those of each other. The study did not commence until all involved were schooled and clear on the intended measurement protocol. This took approximately six 1-h sessions over a 6-week period. Reviewers were blinded to each other’s measurements and their own between clinical and reformatted measurements until the data collection was complete.

At the time of image analysis and version measurement, each reviewer created a screenshot showing their image slice selection, the position of their reference axes, and numerical measurements. These screenshots were used in a preliminary data analysis in which individual measurements that were equal to or greater than one standard deviation from the mean were reviewed. Calculation errors were corrected. All measurements were considered valid as the reviewer could justify their decisions in the placement of the anatomical reference points chosen to create reference lines for version measurement. Patients were scanned in a General Electric Medical Systems LightSpeed VCT Scanner (General Electric, Fairfield, CT, USA). Patients were placed in standard supine position but due to the fracture, were in a sling and therefore the distal humerus was internally rotated. Axial images were obtained with 0.6-mm slices collimation. Images were stored in DICOM format for further processing.

Image analysis and version measurement: The first decision made in the measurement of version is which image slice to use for analysis [1, 2, 5, 19]. Our pilot work found the mid-glenoid level instruction to be inconsistent so we added requirements to the image selection process. Reviewers were instructed to use the slice at the mid glenoid position either by counting the number of slices that included the glenoid and taking the middle one. In our pilot work, this often led to uncertainty as to which slice contained the absolute top and bottom of the glenoid yielding a different “middle slice”. We added the further instruction of scrolling through the several candidate slices in the middle choosing the one that showed the greatest glenoid girth free from artifact and distortion. Numerous things that are difficult to articulate like labral calcifications, small osteophytes on the glenoid rim, occasional CT artifact can alter one’s choice of points from which to draw the line representing the anterior and posterior corners of the glenoid. Additional requirements were that this slice was always inferior to the base of the coracoid and allowed for clear visualization of the medial border of the scapula. The image slice selection chosen by the reviewer was recorded as part of the analysis.

**Fig. 1:**
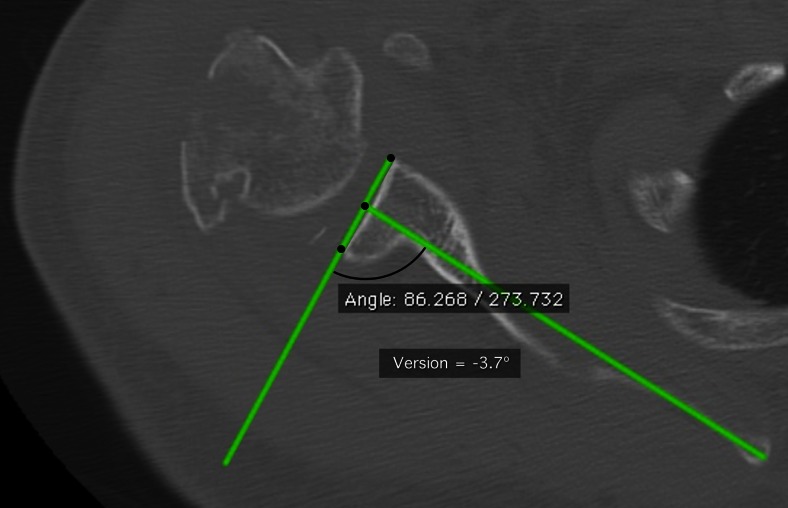
The axial image of subject 18 selected for measurement by reviewer C. Retroversion angle is the angle subtended by the glenoid line and the perpendicular to the scapular line

Placement of reference axes: Reference axes were chosen as conventionally described, one (the scapular line) from the medial border of the scapula through a line connecting the anterior and posterior corners of the glenoid (the glenoid line) at the measured midpoint between the two (Fig. 1).

Measurement of version: Retroversion was measured as the angle subtended by the glenoid line and a perpendicular to the scapular line on the posterior aspect of the scapula. Retroversion was assigned negative values, anteversion positive values. Clinical version was measured in the manner described on the axial (transverse) images, as they were delivered from the technician through the picture archiving and communication system (PACS) (Fig. 1). For anatomic version, these measurements were made after the reformatting protocol described below.

**Fig. 2:**
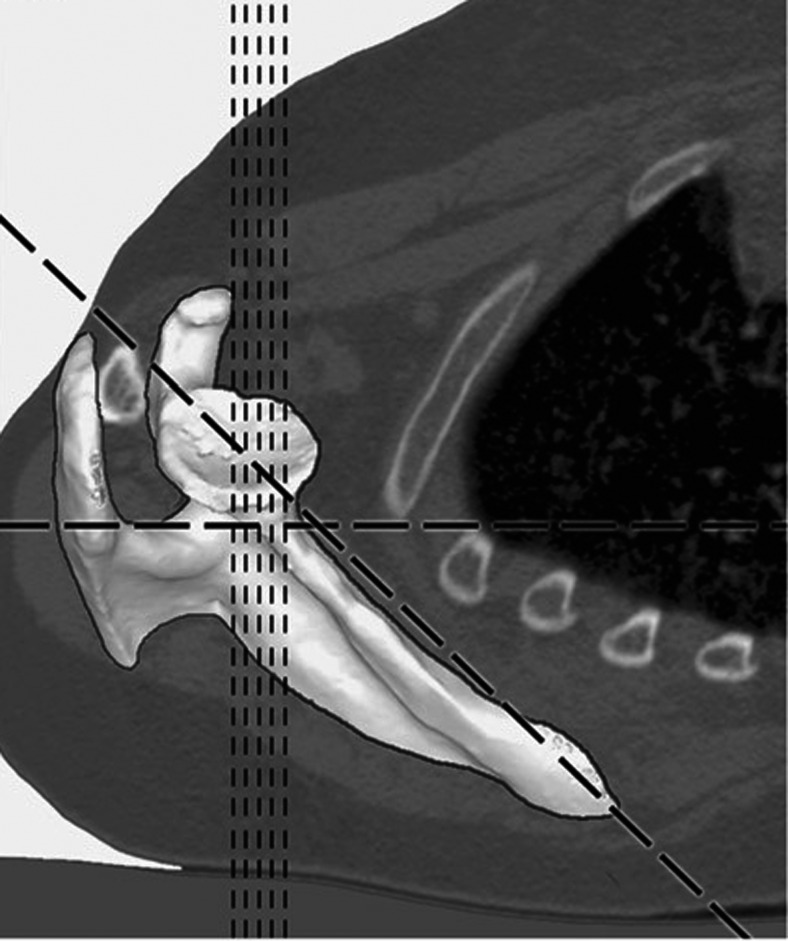
The difference between the orientation of standard axial 2D-CT scans (*vertical lines*) and the orientation of the scapula (*diagonal line*). Reprinted from: Hoenecke HR, Hermida JC, Flores-Hernandez C, D’Lima DD. Accuracy of CT-based measurements of glenoid version for total shoulder arthroplasty. J. Shoulder Elbow Surg. 2010;19:166–171, with permission from Elsevier

**Fig. 3:**
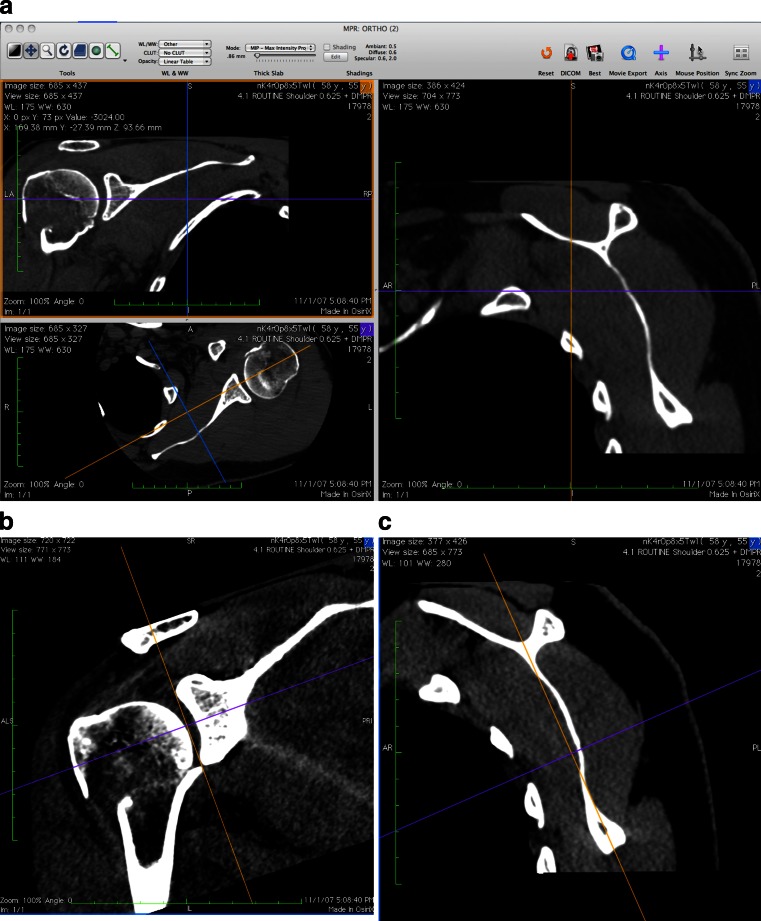
**a** Screenshot from Osirix software showing multi-planar reformatting (MPR) interface that allows reconfiguration of coronal, sagittal, and axial axes. Three orientations are shown simultaneously, coronal on the* top left*, transverse on the* bottom left* and sagittal on the* right*. **b** Screenshot in Osirix software showing reformatted coronal axis aligned with superior-inferior axis of glenoid. **c** Screenshot in Osirix software showing reformatted sagittal axis aligned with mid scapular body

Reformatting protocol: Clinical measurements are made on two-dimensional projections taken in the plane of the body (i.e., the thorax) (Fig. 2). The goal of reformatting is to realign reference axes so that the coronal, transverse, and sagittal planes relate to the scapula and the glenoid (Fig. 3a). Using three-dimensional models, the choices are almost unlimited. Bryce et al. used a coordinate system based on a combination of scapular points and the mid point in the glenoid. Hoenecke et al. constructed a coordinate system from the superior/inferior axis of the glenoid face. Clinical tools presently have greater limitations than models in that not all anatomic points are simultaneously accessible. Through multiple iterations, we evolved a protocol that oriented the glenoid in relation to the scapular plane as best represented by the scapular body. Three reviewers used the General Electric Advantage Workstation with Version 4.4 reformatting software. One reviewer working on a Macintosh computer (Mac OS X; Apple Computer, Cupertino, CA, USA) used an open-source DICOM compatible software OsiriX for reformatting and measurements.

On the transverse plane view, the data set is scrolled to an image below the coracoid to best represent the mid-glenoid. The reference axes are aligned so that one of them lies as best as possible along the surface of the glenoid face. Next, in the coronal plane view, a reference axis is lined up so that it connects the superior and inferior aspects of the glenoid (Fig. 3b). Next, while in the sagittal plane view, scrolling back and forth as necessary until the scapular body is best understood, a slice that best shows the orientation of the scapular body is identified. Then, the final reference axis is set parallel to the orientation to the scapular body (Fig. 3c).

Finally, returning to the transverse plane view, the images were exported for version measurements. The appropriate image slice was chosen and version was measured as described for the measurement of clinical version. Reformatting alters the image slice sequence after which the orders are not directly comparable between reviewers so it was not recorded.

The intraclass correlation coefficient (ICC) for the four raters was calculated according to the method of Shrout and Fleiss using their ICC (2, 1) model [20]. The estimate of the intraclass correlation coefficient is calculated, using SAS, from the mean squares calculated in two-way random effects analysis of variance (ANOVA) models of clinical (as measured) version angle, and of anatomic (reformatted) version angle on patients and reviewers (PROC General Linear Model).

## Results

The mean clinical version from all reviewers was −1.4° ± 6.3°, range, −13.5° to 11.3° (Fig. 1). The maximum retroversion measurement by a single reviewer was −16°, and the maximum anteversion measurement was 21° (Table 1). The inter-observer reliability by ICC was 0.70, *p* < .0001.

**Table 1:** Clinical version

CT subject	Reviewer				All by subject				
	A	B	C	D	Mean	SD	Min	Max	Range
1	−2	2	−7	−2	−2.3	3.8	−7	2	9
2	9	12	12	12	11.3	1.5	9	12	3
3	−2	−2	−3	−2	−2.3	0.3	−3	−2	1
4	−12	−10	−16	−16	−13.5	2.9	−16	−10	6
5	1	0	−1	1	0.3	0.9	−1	1	2
6	−8	−11	−14	−12	−11.3	2.7	−14	−8	6
7	−1	−3	−3	−3	−2.5	1.0	−3	−1	2
8	−3	1	4	6	2.0	3.9	−3	6	9
9	1	−3	−2	−1	−1.3	1.8	−3	1	4
10	−6	−3	−6	−7	−5.5	1.7	−7	−3	4
11	4	0	3	−1	1.5	2.3	−1	4	5
12	−3	−2	−1	−3	−2.3	1.2	−3	−1	2
13	21	6	−6	−10	2.8	13.9	−10	21	31
14	1	4	−2	3	1.5	2.7	−2	4	6
15	−15	−13	−15	−11	−13.5	1.9	−15	−11	4
16	9	0	5	3	4.3	3.8	0	9	9
17	−4	−4	−5	−5	−4.5	0.5	−5	−4	1
18	0	−3	−4	2	−1.3	2.7	−4	2	6
19	2	−3	3	3	1.3	2.8	−3	3	6
20	11	7	4	10	8.0	3.3	4	11	7
Mean	0.2	−1.3	−2.7	−1.7	−1.4	2.8	−4.4	1.8	6.2
SD	8.1	6.0	7.0	7.2	6.3	2.8	6.0	7.7	6.4
Min	−15.0	−13.0	−15.5	−16.0	−13.5	0.5	−16.0	−11.0	1.0
Max	21.0	12.0	11.9	12.0	11.3	13.9	9.0	21.0	31.0
Range	36.0	25.0	27.4	28.0	24.8	13.4	25.0	32.0	30.0

**Fig. 4:**
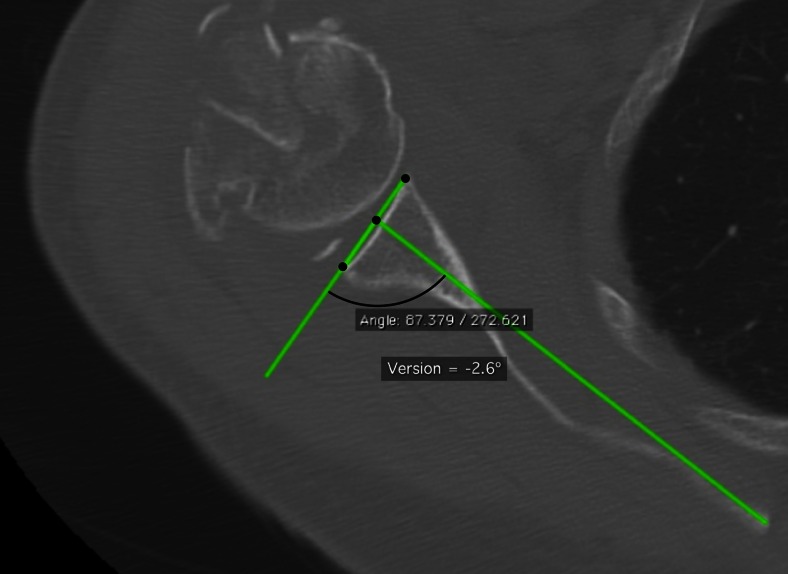
Anatomic (reformatted) axial image of subject 18 (same subject as in Fig. 1), as measured and reformatted by same reviewer (C)

The mean anatomic version from all reviewers was −3.2° ± 4.0°, range, −12.3° to 5.0° (Fig. 4). The maximum anatomic retroversion measurement by a single reviewer was −21°, and the maximum anteversion measurement was 6° (Table 2). The inter-observer reliability by ICC was 0.66, *p* < .0001.

**Table 2:** Anatomical version

CT subject	Reviewer				All by subject				
	A	B	C	D	Mean	SD	Min	Max	Range
1	−2	−5	−4	−2	−3.3	1.5	−5	−2	3
2	0	1	−3	−4	−1.5	2.4	−4	1	5
3	−3	−6	−4	−4	−4.3	1.3	−6	−3	3
4	−5	−21	−7	−5	−9.5	7.7	−21	−5	16
5	−1	−4	0	−5	−2.5	2.4	−5	0	5
6	0	−4	−1	−5	−2.5	2.4	−5	0	5
7	−4	−3	0	1	−1.5	2.4	−4	1	5
8	−7	−7	−4	−5	−5.8	1.5	−7	−4	3
9	−2	−6	−4	1	−2.8	3.0	−6	1	7
10	−4	−7	−4	−5	−5.0	1.4	−7	−4	3
11	3	0	2	0	1.3	1.5	0	3	3
12	1	−1	2	1	0.8	1.3	−1	2	3
13	−5	−4	−8	−5	−5.5	1.7	−8	−4	4
14	3	6	5	6	5.0	1.4	3	6	3
15	−15	−10	−16	−8	−12.3	3.9	−16	−8	8
16	−12	−10	−9	−6	−9.3	2.5	−12	−6	6
17	−2	−5	−3	−5	−3.8	1.5	−5	−2	3
18	0	0	−3	2	−0.3	2.1	−3	2	5
19	−2	−4	−2	−4	−3.0	1.2	−4	−2	2
20	5	−3	−1	3	1.0	3.7	−3	5	8
Mean	−2.6	−4.7	−3.2	−2.5	−3.2	2.3	−6.0	−1.0	5.0
SD	4.8	5.4	4.5	3.7	4.0	1.5	5.3	3.7	3.1
Min	−15.0	−21.0	−16.0	−8.0	−12.3	1.2	−21.0	−8.0	2.0
Max	5.0	6.0	5.0	6.0	5.0	7.7	3.0	6.0	16.0
Range	20.0	27.0	21.0	14.0	17.3	6.6	24.0	14.0	14.0

The mean difference between clinical and anatomic version across all subjects from all reviewers was 1.9° ± 5.6°, range, −8.8° to 13.5° (Tables 3).

**Table 3:** Version angle differential

CT subject	Reviewer				All by subject				
	A	B	C	D	Mean	SD	Min	Max	Range
1	0	7	−3	0	1.0	4.2	−3	7	10
2	9	11	15	16	12.8	3.3	9	16	7
3	1	4	1	2	2.0	1.4	1	4	3
4	−7	11	−9	−11	−4.0	10.1	−11	11	22
5	2	4	−1	6	2.8	3.0	−1	6	7
6	−8	−7	−13	−7	−8.8	2.9	−13	−7	6
7	3	0	−3	−4	−1.0	3.2	−4	3	7
8	4	8	8	11	7.8	2.9	4	11	7
9	3	3	2	−2	1.5	2.4	−2	3	5
10	−2	4	−2	−2	−0.5	3.0	−2	4	6
11	1	0	1	−1	0.3	1.0	−1	1	2
12	−4	−1	−3	−4	−3.0	1.4	−4	−1	3
13	26	10	2	−5	8.3	13.3	−5	26	31
14	−2	−2	−7	−3	−3.5	2.4	−7	−2	5
15	0	−3	1	−3	−1.3	2.1	−3	1	4
16	21	10	14	9	13.5	5.4	9	21	12
17	−2	1	−2	0	−0.8	1.5	−2	1	3
18	0	−3	−1	0	−1.0	1.4	−3	0	3
19	4	1	5	7	−4.3	2.5	1	7	6
20	6	10	5	7	7.0	2.2	5	10	5
Mean	2.8	3.4	0.5	0.8	1.9	3.5	−1.6	6.1	7.7
SD	8.2	5.4	6.8	6.6	5.6	3.1	5.6	8.0	7.0
Min	−8.0	−7.0	−13.0	−11.0	−8.8	1.0	−13.0	−7.0	2.0
Max	26.0	11.0	15.0	16.0	13.2	13.3	9.0	26.0	31.0
Range	34.0	18.0	28.0	27.0	22.3	12.4	22.0	33.0	29.0

Using the stated image slice selection criteria, reviewers differed in their slice selection by a mean of 7.7 slices per subject (range, 3–15). In no instance did all four reviewers select the same image slice to make their measurements. For only one subject did three of four reviewers choose the same slice. For five subjects, the reviewers chose all different slices from each other. For the remaining 14 subjects, two of four reviewers chose the same slice.

## Discussion

This study demonstrated reasonable inter-observer variability at multiple levels but highlights ways in which variability permeates the clinical experience. After correction for scapular orientation, the range of version values observed in this study decreased from 24.8° (−11.3° to +13.5°) to 17° (−4.9° to +12.3°) and the standard deviation decreased from 6.3° to 4°. Bryce et al. also observed this reduced range of version values in their detailed study of scapular models made from cadavers. The version range measured by these authors decreased from 21.1° (−8.5° to +22.6°) to 15.4° (−8.8 to +7.6°) after correcting for scapular orientation. Of note, their standard deviation also decreased from 8.0° to 3.8° between clinical and anatomic values [6]. Hoenecke et al. found an average variation between clinical version and anatomic version of 5.1° but as high as 16° [21]. This contracted range of values suggests that some of the variability previously reported for glenoid version has been due to inconsistencies in methods of image acquisition. As addressed in the Results section, our mean clinical version was −1.4° ± 6.3° (range, −13.5° to 11.3°), and for the anatomic version −3.2° ± 4.0°, (range, −12.3° to 5.0°). Compared with Bryce et al. where they measured an average clinical version of 3.8° ± 8.0° (range, −8.5° to 22.6°) and −2.0° ± 3.8° (range, −8.8° to 7.6°) of anatomic version, using their 3D model. Hoenecke et al. measured a mean anatomic version of −8.6° ± 9.8°. As can be seen by these compared results, the mean values change slightly but are consistent.

Inter-observer reliability in the measurement of version on axial CT images has been evaluated by others with ICC values generally better than ours ranging from 0.79 to 0.97 [7, 22, 23]. We can only account for these differences by relating the measurement circumstances of our own study that included a training period, three reviewers who were radiologists at varying levels of experience and an orthopedic surgeon with specialty in shoulder surgery. Furthermore, models offer greater control over the reference points selected and one of these studies was a cadaveric study that was corrected for scapular orientation through a holding device on the 2D images. Two recent analogous studies evaluated inter-observer reliability of three reviewers measuring foot angles from radiographs publishing values consistent with ours, ICC values typically in the range from 0.60 to 0.80 [24, 25]. Radler et al. cautioned that although the measurements were more reliable than they expected, “measurement discrepancies can be as great as 30°” [24].

The clinical implications of this study relate to any discussion or technique that aims to restore glenoid version. Without reproducible methods to define the anatomy or a sense of the error in existing methods, specifying surgical goals to strive for a specific value of version are unfounded. Surgical techniques aiming to correct or “normalize” glenoid version have justification from the biomechanical literature and have been the focus of several recent studies. Technology for computer navigation to do the same is under development in several centers [26]. It is clear from the forgoing discussion, however, that these efforts will be incapable of achieving their goal without rigorous methodology for measuring glenoid version pre-operatively and intra-operatively. Furthermore, they will not be able to gauge their effectiveness without applying the same rigor post-operatively.

With the software used at the time of this study, the ability to manipulate the 3D anatomy was limited but it had the advantage of being clinically accessible, even free on the Macintosh platform. We used two platforms in this study primarily to improve the accessibility of our protocol for manipulating the three-dimensional anatomy of the scapula by adjusting its spatial orientation from a series of 2D images. For the coronal and transverse planes, the superior/inferior and anterior/posterior edges of the glenoid were readily identifiable as they would be with a 3D model. Defining the sagittal plane in line with the scapular body required the most subjectivity, as the scapular body is usually curved and rarely sits nicely in a plane. The practice of scrolling back and forth through the 2D images medial to the glenoid allowed us to approximate the scapular body axis but this step admittedly would benefit from greater precision. Despite these limitations, this multi-step process only marginally diminished ICC values—a worthwhile tradeoff for improved angular measurements.

Image slice selection is the first step in the measurement process. The established, and often quoted, practice of measuring version at the middle glenoid level by CT scan has its origin in the first papers on this subject when the technology allowed for cuts every 5–10 mm. Modern-day scanners allow for sub-millimeter cuts (0.63 mm in this study) meaning for the average 40-mm glenoid, at least ten slices that may appear suitable. Depending on how one defines the absolute top and bottom, even specifying the exact middle creates some degree of indeterminacy. Other factors then come into play in the selection of the “best” slice for measurement, such as how well the anterior and posterior edges are visualized (possibly obscured by CT artifact, labral calcifications, glenoid rim osteophytes), or whether the medial border of the scapula is well seen. It is our contention that these factors in version measurement have not been reconsidered in the context of modern imaging technology. Our observations of multiple examiners confirm this although admittedly do not yet offer a methodological solution.

As a study intending to explore aspects of how we measure glenoid version by CT scan in a clinical setting, this study has notable limitations. For practical reasons, we did not exhaustively study all components of the process. For example, we identified variability in image slice selection between reviewers but we did not look at the variance in version that results from slice to slice. We also identified variability in the selection of reference points from which the axes for the glenoid and scapular lines are determined. Here lie opportunities for future research in determining which of these factors contribute most to intra-patient variance in measuring glenoid version. Different platforms were used to perform our measurements, possibly adding to variability, although we did not discover such a pattern after our statistical analysis. This study did not look at intra-observer variability, expecting that it exists albeit perhaps to a lesser extent than inter-observer variability. The concerns of this investigation were focused on the variability between observers related to technical considerations of the CT scanning process.

The version values observed in this study, both clinical and anatomic, were consistent with those in the literature—essentially near zero—reinforcing the notion that the glenoid face is normally perpendicular to the scapular plane. The precise value of version is dependent on the chosen definition of the scapular plane. Some variance between our measures of version and those recently reported in the studies using 3D models is expected as we used slightly different reference axes to define the version angle. For example, in the methods described by Bryce et al., the coronal plane is defined by the medial border of the scapula and the central point of the glenoid fossa [6]. We used the superior-inferior axis of the glenoid to orient the coronal plane. Assuming an upward tilt to most glenoids, we measured version in an adducted position relative to their coordinate system; and in their study, abduction/adduction introduced the greatest variability in version measurements as compared to rotation about other axes.

The use of 3D models by Bryce et al., Hoenecke, and others [6–8, 21] has greatly informed our understanding of scapular anatomy, and is recommended by these authors as the method best suited for this kind of study. Models are superior to the methodology we employed and at this juncture. Models are superior in that one has greater control over the reference points selected to define the scapular and glenoid planes, and therefore reference axes for measurement. They are not, however, without limitations. Models require specialized software that is expensive and requires some expertise in its use. Furthermore, once built, 3D models do not eliminate user variability in that one must still assign reference points for axis determination making them subject to user discretion and error.

## Conclusions

This study confirmed the variation in glenoid version dependent on scapular orientation previously identified in other studies using scapular models, and presents a clinically accessible protocol to correct for scapular orientation from the patient’s CT data. Future clinical software will likely make this CT scanning process more practical and reproducible.
